# Cohort Effects in Alzheimer’s Disease Trials: An Empirical Assessment Utilizing Data from the Alzheimer’s Disease Cooperative Study

**DOI:** 10.14283/jpad.2023.45

**Published:** 2023

**Authors:** A.I. Birnbaum, J.D. Grill, D.L. Gillen

**Affiliations:** 1. Department of Statistics, University of California, Irvine, Irvine, CA, USA; 2. Institute for Memory Impairments and Neurological Disorders, University of California, Irvine, Irvine, CA, USA; 3. Alzheimer’s Disease Research Center, University of California, Irvine, Irvine, CA, USA; 4. Department of Neurobiology and Behavior, University of California, Irvine, Irvine, CA, USA; 5. Department of Psychiatry and Human Behavior, University of California, Irvine, Irvine, CA, USA

**Keywords:** Alzheimer’s Disease, cohort effects, clinical trial design

## Abstract

**BACKGROUND:**

Cohort effects in study populations can impact clinical trial conclusions and generalizability, particularly in trials with planned interim analyses. Long recruitment windows may exacerbate these risks in Alzheimer’s disease (AD) trials.

**OBJECTIVES:**

To investigate the presence of cohort effects mild-to-moderate AD trials.

**DESIGN:**

Retrospective analysis using pooled participant-level data from nine randomized, placebo-controlled trials conducted by the Alzheimer’s Disease Cooperative Study (ADCS).

**SETTING:**

Trials were multicenter studies conducted by an academic trial network.

**PARTICIPANTS:**

The trials enrolled participants with mild, mild-to-moderate, or moderate AD who were over age 50 and had mini mental state exam scores between 12 and 26.

**INTERVENTIONS/EXPOSURE:**

We defined a participant’s site-standardized enrollment time as the number of days between their screening date and the first screening date among randomized participants at their site within their study.

**MAIN OUTCOME(S) AND MEASURE(S):**

Our primary outcome was the 12-month change in the AD assessment scale – cognitive subscale (ADAS-Cog). Secondary outcomes were participant demographics and time to study discontinuation.

**RESULTS:**

The pooled sample consisted of N=2,754 at baseline with N=2,191 participants completing a 12-month visit. We found no meaningful differences in the distributions of sex, race and ethnicity, age, years of education or baseline ADAS-Cog score across enrollment time. We found a significant association between enrollment time and 12-month change in ADAS-Cog, with participants enrolling 100 days later tending to experience an increase on the ADAS-Cog of 0.16 points greater (reflecting greater cognitive decline; 95% CI: (0.021, 0.294), p = 0.02), after controlling for potential confounding factors.

**CONCLUSION:**

We found minimal evidence of clinically relevant cohort effects in ADCS trials. Our results reinforce the original findings of these trials.

## Introduction

Clinical trials rely on well-defined patient populations to draw meaningful and generalizable conclusions about treatments under study. Recruiting a sample of participants that is representative of the target population and instituting proper randomization are key to avoiding bias in estimates of treatment effects. These processes can be disrupted by cohort effects; if participants who come into trials later are different from those enrolled earlier. Shifts in participant characteristics over the course of accrual may yield misleading estimates of treatment effects at interim analyses and/or potential bias due to imbalances of confounding factors across treatment groups.

Alzheimer’s disease (AD) trials are at particular risk for biases from cohort effects due to extended recruitment windows that result from high rates of exclusion and logistical barriers to participation (1, 2). Participants recruited later into trials may differ from those recruited earlier due depletion of pools of readily available participants or due to differential recruitment rates across heterogeneous study sites (3).

The implications of cohort effects recently came to light in the controversial EMERGE and ENGAGE trials of the anti-amyloid treatment aducanumab. In these trials, the sponsor contended that a cohort effect resulted in an invalid interim futility analysis and inappropriate trial stoppage (4). This situation exemplifies that the presence of cohort effects can have critical repercussions on trials and their findings.

Here, we aimed to assess the potential presence of cohort effects in a set of nine AD trials performed by the Alzheimer’s Disease Cooperative Study (ADCS), a joint initiative between academic trialists and the National Institute on Aging. In particular, we investigated whether cohort effects were present with respect to demographic data, cognitive decline, study partner type, and study discontinuation.

## Methods

### Data Source

We used data from nine ADCS trials that were conducted between 1999 and 2014. We received the datasets through the University of California, San Diego ADCS Legacy database and the Alzheimer’s Therapeutic Research Institute at the University of Southern California. In chronological order, the study interventions tested in the ADCS trials were the non-steroidal anti-inflammatory drugs (NSAIDs) rofecoxib and naproxen (5), simvastatin (6), high dose vitamin B supplementation (7), valproate (8), huperzine A (9), docosahexaenoic acid (DHA) (10), intravenous immunoglobin (IVIg) (11), resveratrol (12), and the FYN kinase inhibitor AZD0530 (13). All trials were placebo controlled and failed to demonstrate superiority of the intervention over placebo on their prespecified primary outcomes.

With the exception of the huperzine A trial, which had a primary endpoint of 16 weeks and an optional extension study that ran for up to 12 additional months, each trial had a duration of at least 12 months. Seven trials enrolled participants with mild-to-moderate AD, one enrolled participants with moderate AD (valproate), and one enrolled participants with mild AD (AZD0350). The trials included similar mini mental state exam (MMSE) ranges in their inclusion criteria. The largest difference was between the valproate (range: 12–20) and AZD0530 (range: 18–26) trials. Four participants had missing race and ethnicity information and one had missing years of education. We omitted these participants from regression analyses. Thus, our combined sample consisted of N=2,754 participants at baseline and 2,191 participants at 12 months. For analyses involving study partner type we omitted the IVIg data which did not include study partner information.

### Defining Time of Enrollment

We defined site-standardized enrollment time as the number of days between a participant’s screening visit and the first screening visit among randomized participants at the same site within the same study. This construction standardizes at the study level, to account for calendar time differences between studies, and at the site level, to account for differences in site time to activation. We chose to standardize at the site level to reflect the hypothesis that cohort effects may be caused by local depletion of eligible patient pools.

In secondary analyses, we defined a participant’s trial enrollment percentile as the proportion of participants enrolled into a given study at the time they enrolled. For example, if a trial’s total sample size was 100 participants, the participant with the 10th earliest screening date is assigned an enrollment percentile of 0.10 since, at that time, 10/100 participants had enrolled. In this construction there is no site-level standardization. We chose this definition to align with sequential testing frameworks where interim analyses are commonly performed when pre-specified proportions of the full sample reach the primary endpoint (14). We primarily treated both site-standardized enrollment time and trial enrollment percentile as continuous predictors in regression analyses.

### Outcome Variables

To assess cohort effects on trial outcomes, we used the 12-month within-subject change in the 11-item ADAS-Cog (15). We chose the 12-month change because all trials had data up to at least that point and each trial recorded the ADAS-Cog at the 12-month visit. If participants had an incomplete task coded as being due to cognitive reasons, we imputed the maximum possible score for that task. If participants had an incomplete task coded as being due to a non-cognitive reason (either participant refusal or due to physical reasons) we imputed their average score among completed tasks. In the IVIg dataset, we were not able to compute participants’ scores on the word recognition task due to lack of word-specific data collection. For these participants we performed hot deck imputation and imputed the score of the participant whose other task scores most closely matched among participants in all other trials; if multiple participants were equally similar, we imputed the average score among these participants. We determined that the marginal distribution of imputed scores was similar to the observed distributions in other trials via visual inspection. Where applicable, we repeated analyses without the IVIg dataset to assess sensitivity to our imputation procedure.

To assess the association between enrollment time and study partner type, we categorized study partners into three groups: spouse, adult child, or other. We did not have study partner information for the IVIg trial, hence this dataset was necessarily omitted for relevant analyses.

We defined a participant’s time to study discontinuation as the number of days between their screening date and the date they dropped out of their study. Withdrawing consent, discontinuing participation due to an adverse event other than death, or being lost to follow-up were all treated as dropping out of the study. If a participant reached their specific study’s primary endpoint (16 weeks, 12 months, 18 months, or 24 months, depending on the study) or died prior to either completing the study or dropping out, we treated them as censored. In addition to treating their time to study discontinuation in days as a continuous measure, we also considered a binary indicator of primary endpoint completion.

### Statistical Analyses

To investigate potential differences in patient baseline characteristics over enrollment time, we split our sample into quartiles by enrollment time and qualitatively assessed for potential differences in distributions. We summarized continuous characteristics using means and standard deviations and summarized categorical characteristics using counts and proportions.

To analyze cohort effects on trial outcomes, we regressed the 12-month change in ADAS-Cog on enrollment time while controlling for baseline score, sex, age, race & ethnicity, and years of education. We included trial as a factor variable in this and all other regression models. We used generalized estimating equations (GEE) (16) with an exchangeable working correlation structure at the study-site level to allow for clustering between participants who attended the same site in the same study. Confidence intervals (CIs) were constructed using robust standard errors (17) unless stated otherwise.

To analyze cohort effects on study partner type, we regressed a participant’s study partner type at baseline on the same covariates as above using multinomial logistic regression. To account for potential clustering at the study-site level, we obtained bootstrap variance estimators for the regression parameter estimates. However, CIs obtained via these bootstrapped estimates did not qualitatively differ from those obtained with model-based variance estimators for any model parameters. Therefore, CIs reported for this analysis use the model-based standard errors.

To analyze cohort effects on participant retention marginally, we used Kaplan-Meier estimates stratified by site-standardized enrollment time quartiles. To analyze cohort effects on participant completion rates while controlling for potential confounding factors, we fit a Cox proportional hazards model with the same aforementioned covariates. To allow for between-trial heterogeneity in baseline hazards, we fit the model stratified by trial. We fit a logistic regression to a binary indicator of whether participants completed their primary endpoint.

All p-values were obtained using Wald tests and all hypothesis tests were 2-sided. The association between the 12-month change in ADAS-Cog and site-standardized enrollment time was our primary inferential target. All other analyses were secondary, and corresponding p-values should be interpreted accordingly. To aid in interpretation and detect potential non-linear trends, we repeated all regression analyses with enrollment time discretized into quartiles and treated as a factor variable. For our secondary investigation, we also repeated all analyses above replacing site-standardized enrollment time with trial enrollment percentile; these results are available in the supplemental content. We performed our analyses using R version 4.2.0 and used the ‘geepack’, ‘nnet’, and ‘survival’ packages for fitting regression models.

## Results

**Figure 1.**
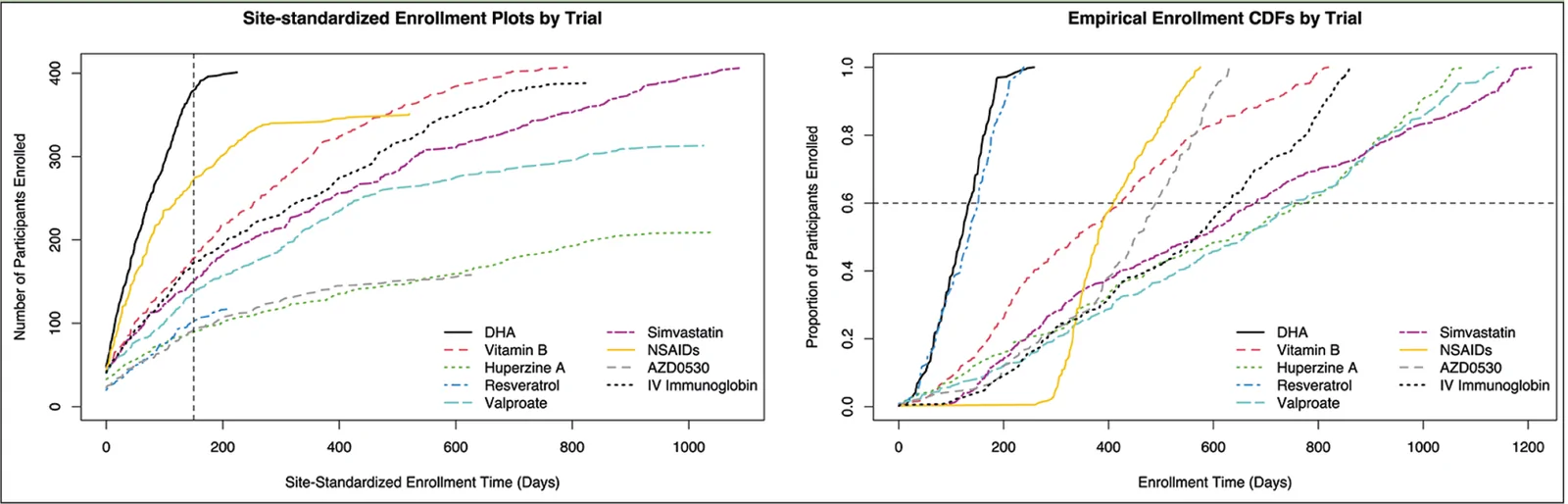
Cohort illustrations Left: Enrollment plots by trial using site-standardized enrollment time; time is standardized at both the trial- and site-level so that for each site within each trial, the time origin represents the earliest screening date among only the subsequently randomized participants at that site; all participants who fall on a given vertical line are assigned an identical site-standardized enrollment time; example dashed line is given at 150 days. Right: Empirical enrollment CDFs by trial using non-site-standardized enrollment time. Time is standardized only at the trial-level so that within each trial, the time origin represents the earliest screening date among all subsequently randomized participants. All participants who fall on a given horizontal line are assigned an identical enrollment percentile; an example dashed line is given at 60% enrollment. CDF, cumulative distribution functions.

Figure 1 displays site-standardized enrollment plots (left panel) and non-site-standardized cumulative distribution functions (CDFs) of enrollment (right panel) for each trial. Of note, all 402 participants in the DHA trial were screened within 224 days of their site activation time. The NSAIDs trial also recruited participants more quickly than the remaining trials, with the vast majority of its 351 participants enrolled within one year. Other trials of similar sample size had sites that took over two years to enroll.

Table 1 presents baseline demographic information, average 12-month change in ADAS-Cog, and percent of participants completing the trial primary endpoint stratified by quartile of site-standardized enrollment time. Empirical enrollment time quartiles were [0,33] days, (33,119] days, (119,322] days and >322 days. Few differences were apparent across these cohorts. Instead, the assessed demographic and clinical characteristics appeared largely uniform. The analogous table stratified by trial enrollment percentile is in Table S1. There was a slight increase in the proportion of participants enrolling with adult child study partners after the first quartile but beyond this, the cohorts appeared largely similar (table S1).

Those in the last enrolled site-standardized quartile did appear to demonstrate greater cognitive decline measured with the ADAS-cog, compared to earlier enrolled participants (Table 1). Using a GEE regression model to assess potential cohort effects with respect to cognitive decline, we found a significant difference in 12-month change in ADAS-Cog for participants with different site-standardized enrollment times (Table 2). Later enrolled participants had an expected 12-month change in ADAS-Cog 0.16 points higher than similar participants who enrolled 100 days earlier (95% CI: (0.02, 0.29), p = 0.02). Analysis using the discretized construct suggested this observed effect may have been primarily due to differences between the earliest and latest enrolling participants: we estimated that participants who enrolled in the fourth quartile of site-standardized enrollment time had an expected 12-month change in ADAS-Cog of 0.97 points higher than similar participants who enrolled in the first quartile (95% CI: (0.03, 1.91), p = 0.04). These results were not sensitive to our imputation procedure as neither of these point estimates were meaningfully changed when omitting the IvIg trial data (though the resulting CIs did narrowly cross over zero due to decreased precision, data not shown). In the analysis using trial enrollment percentile, the sign of point estimates was the same but no coefficients were significantly non-zero (table S2).

**Figure 2.**
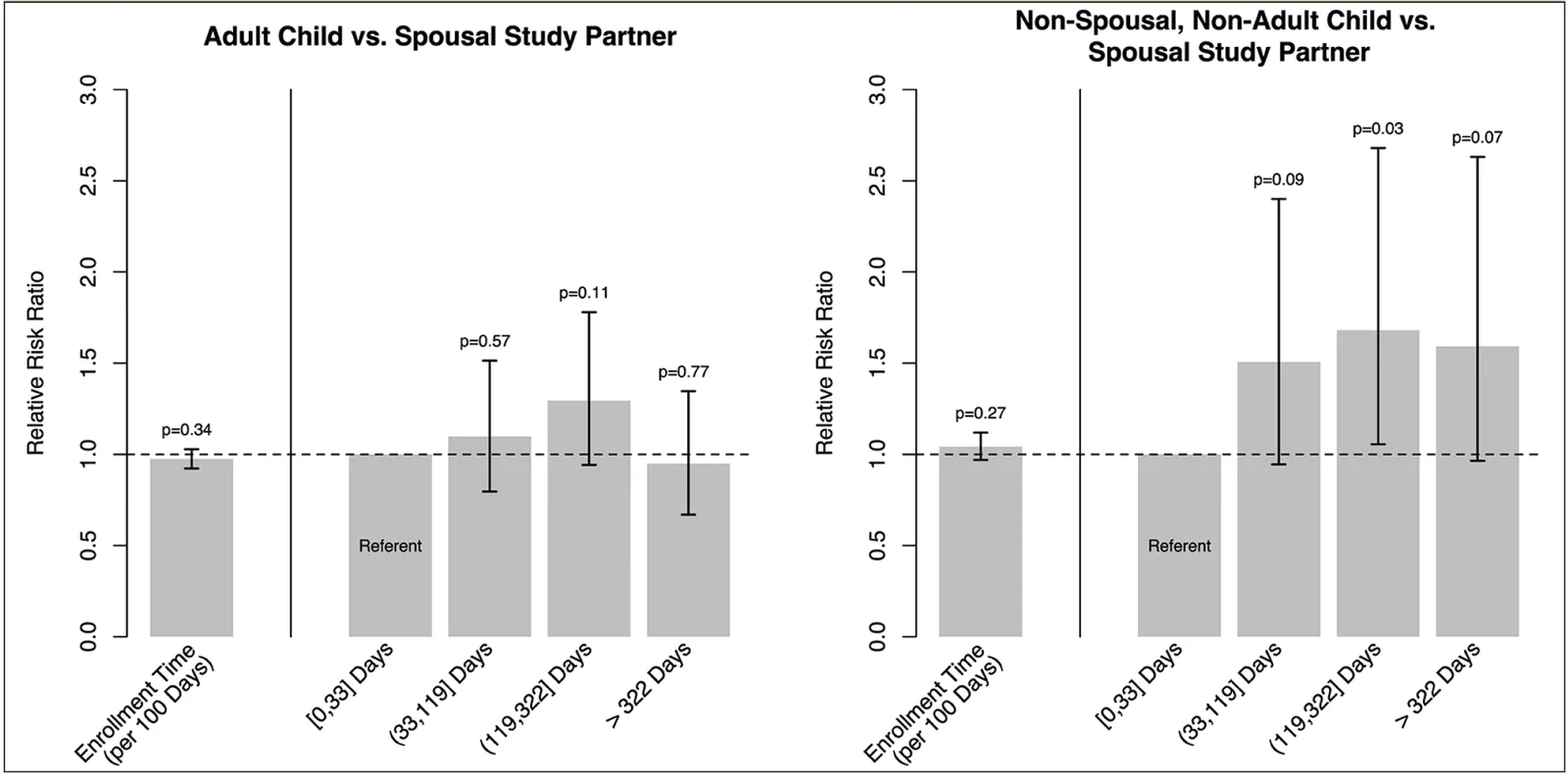
Summary of multinomial regression of study partner type on site-standardized enrollment time Left: summary of relative risk ratios of enrolling with an adult child study partner compared to a spousal one. Right: summary of relative risk ratios of enrolling with an other study partner compared to a spousal one. Within each panel the estimate corresponding to the model using continuous enrollment time is given on the left and those from the model using site-standardized enrollment time quartiles is given on the right.

Figure 2 summarizes the estimated relative risk ratios, 95% CIs, and p-values from the multinomial regression assessing potential cohort effects on study partner type at baseline using site-standardized time. When treated as continuous, there was no meaningful association between enrollment time and the relative risk of enrolling with an adult child study partner compared to a spousal study partner; this was also the case between enrollment time and the relative risk of enrolling with an other study partner compared to a spousal study partner. Analyses using the discretized construction of site-standardized enrollment time did provide some evidence that later enrolled participants had a higher relative risk of enrolling with an other study partner compared to the first quartile reference group (right side of right panel of figure 2, top). We estimated that participants who enrolled in the 2nd, 3rd, and 4th quartiles had 50, 68, and 59% higher relative risk of enrolling with an other study partner type, compared to a spousal one, than similar participants who enrolled in the 1st quartile (95% CI for 2nd quartile relative risk (0.94, 2.40), p = 0.09; for 3rd quartile: (1.06, 2.68), p = 0.03; for 4th quartile: (0.96, 2.63), p = 0.07). In the secondary analysis using trial enrollment quartiles, we found evidence that later enrolled participants had a higher relative risk of enrolling with an adult child study partner than with a spousal one compared to the first quartile reference group (95% CI for 2nd quartile relative risk ratio: (1.12, 2.16), p = 0.009; for 3rd quartile: (1.10, 2.11), p = 0.01; for 4th quartile: (1.04, 2.00), p = 0.03; figure S1).

**Figure 3.**
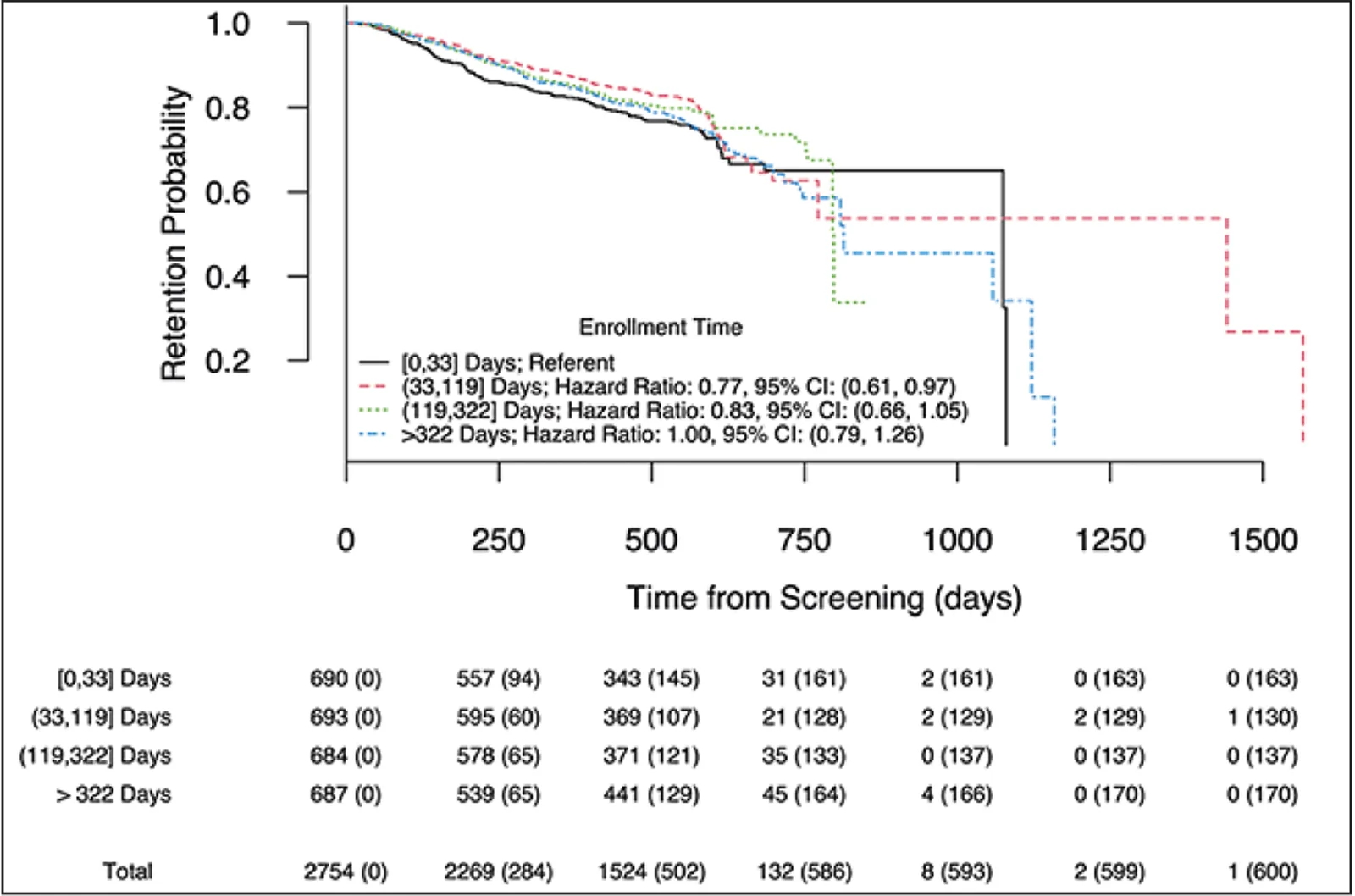
Kaplan-Meier curves by site-standardized enrollment time quartiles Legend: corresponding hazard ratio estimates and 95% CIs from a Cox proportional hazards model of time to study discontinuation on discretized site-standardized enrollment time; study completion or dying prior to primary endpoint completion were considered censoring. Bottom: Number of subjects at risk; cumulative number of dropout events given in paratheses.

Figure 3 illustrates Kaplan-Meier curves estimating the retention probability over study duration, stratified by site-standardized enrollment time quartiles. Retention probability was similar across the cohorts over the duration where we had relatively high precision (up to around 18 months). We found no significant association between enrollment time and hazard for discontinuation when treating enrollment time as continuous (estimated hazard ratio for an enrollment time difference of 100 days: 1.02, 95% CI: (0.99, 1.07), p = 0.16). Estimated hazard ratios from the model using discretized site-standardized enrollment time can be seen in the legend of figure 3. In this model, participants who enrolled in the 2nd quartile had significantly lower hazard for study discontinuation compared to similar participants who enrolled in the first quartile (95% CI: (0.77, 0.97), p = 0.03), though the trend did not continue for subsequent enrollment time quartiles. Secondary analysis using trial enrollment quartiles similarly displayed little evidence of cohort effects with respect to hazard for study discontinuation (figure S2). The results of the logistic regression with binary study completion status as the outcome (not shown) similarly suggested minimal cohort effects with respect to early study discontinuation.

## Discussion

The financial and human resource costs of clinical trials emphasize the imperative to ensure the validity and generalizability of findings. Identifying and addressing sources of bias in downstream estimates of treatment effects represents a key aspect of this effort. Cohort effects can lead to bias in at least two ways. First, heterogeneity in participant characteristics over the duration of accrual could lead to imbalances of confounders of the target treatment effect across study arms. Second, if an effect-modifier of the treatment effect varies with enrollment time, study results could be biased or even erroneous. Although balance across treatment groups may be restored by chance (in the first case) or we may obtain a representative sample of the target population by the end of accrual (in the second case), each of these scenarios can yield biased estimates and the appearance of a non-constant treatment effect across interim analyses. Potential treatment group imbalances due to cohort effects can be avoided (in both interim and final analyses) by block randomizing (18), which should be implemented whenever feasible.

In this analysis we investigated whether cohort effects were present in a combined sample of nine trials performed in participants with mild to moderate AD. We found no clinically meaningful differences across site-standardized enrollment time or trial enrollment percentile with respect to participant demographics, baseline ADAS-Cog score, or dropout. This is good news. A lack of cohort effects lends further credence to the conclusions reported for these trials.

We did find evidence that later-enrolled participants tended to experience slightly larger cognitive decline over the first 12 months of trial participation. Discretizing site-standardized enrollment time into quartiles suggested the observed difference was primarily driven by heterogeneity between the earliest- and latest-enrolling participants. Although these differences were statistically significant, the effect sizes were small and thus may not be clinically relevant. For example, many AD trials, including some included here, are powered to demonstrate around a 2-point difference between groups on the ADAS-cog; our observed difference was only 8% of this difference.

When modeled as a continuous variable, we found no association between enrollment time and study partner type at baseline. When discretized into quartiles, however, we estimated that participants enrolling in any quartile after the first had higher relative risk of enrolling with a non-spousal, non-adult child study partner than with a spouse, compared to participants enrolling in the first quartile. Though we saw no cohort effects for the outcome of dropout, previous studies have found that these participants, who enroll with a study partner that is neither their spouse nor their adult child, may be at increased risk for dropout compared to those with a spouse (19). Minority race and ethnicity participants may also more frequently enroll with these study partners (19–21) perhaps indicating the need for greater time to recruit these underrepresented groups (though we observed no such cohort effect for race or ethnicity). The analysis using trial enrollment percentile suggested an analogous trend with adult child study partners. Elucidating these relationships will require further research but may be important to future trial recruitment.

Our study had limitations. We investigated two constructions of participants’ time of enrollment but other relevant constructs may exist. We attempted to control for potential confounders between enrollment time and our outcome variables, but the target association between variables of interest could have been masked by confounding factors not included in our analyses. We focused on demographic and cognitive outcomes; future analyses might investigate whether temporally non-constant treatment effects occur, especially across interim estimates in AD trials. We did not account for the possibility that cohort effects could have been masked by trials competing to enroll. Finally, although our sample size was large and these trials enrolled participants from throughout the US, our observations may not generalize to AD clinical trials sponsored by industry or performed outside of the US.

## Conclusions

We found minimal evidence of clinically relevant cohort effects in AD trials. Overall, our results bolster the original conclusions of the trials used in our sample.

## Supplementary Material

Supplementary material

## Figures and Tables

**Table 1.** Sample baseline demographics, 12-month change in ADAS-Cog, and study completion percentage stratified by site-standardized enrollment time quartiles

	Site-Standardized Enrollment Time			
	Quartile 1	Quartile 2	Quartile 3	Quartile 4
Enrollment Window	[0,33] Days	(33,119] Days	(119,322] Days	>322 Days
Sex, No. (%)				
Female	380 (55.1)	365 (52.7)	398 (58.2)	391 (56.9)
Male	310 (44.9)	328 (47.3)	286 (41.8)	296 (43.1)
Age, No. (%)				
[50,60)	50 (7.3)	45 (6.5)	46 (6.7)	63 (9.2)
[60,70)	135 (19.6)	137 (19.8)	135 (19.7)	138 (20.1)
[70,80)	293 (42.5)	305 (44.0)	278 (40.6)	262 (38.1)
[80,90)	204 (29.6)	201 (29.0)	210 (30.7)	210 (30.6)
90+	8 (1.2)	5 (0.7)	15 (2.2)	14 (2.0)
Race and Ethnicity, No. (%)				
Non-Hispanic White	614 (89.0)	634 (91.5)	587 (85.8)	610 (88.8)
Non-Hispanic Black	35 (5.1)	31 (4.5)	44 (6.4)	37 (5.4)
Hispanic	28 (4.1)	20 (2.9)	32 (4.7)	36 (5.2)
Asian/Pacific Islander	6 (0.9)	7 (1.0)	9 (1.3)	4 (0.6)
Other	7 (1.0)	1 (0.1)	12 (1.8)	0 (0)
Study Partner Type^a^, No. (%)				
Spouse	445 (72.2)	439 (70.9)	377 (64.0)	363 (67.1)
Adult Child	136 (22.1)	129 (20.8)	159 (26.7)	128 (23.7)
Other	35 (5.7)	51 (8.2)	53 (9.0)	50 (9.2)
Years of Education, mean (SD)	14.39 (3.2)	14.51 (3.0)	14.19 (3.1)	14.39 (3.4)
Baseline ADAS-Cog, mean (SD)	24.76 (9.4)	23.48 (9.6)	24.15 (9.8)	25.49 (10.2)
12-Month ADAS-Cog Change, mean (SD)	5.83 (7.8)	5.38 (7.5)	5.8 (7.3)	6.63 (7.2)
% Completing Primary Endpoint	74.1	78.8	76.8	73.2

a. does not include participants from the IVIg trial

**Table 2.** Summary of GEE regression of 12-month change in ADAS-Cog on site-standardized enrollment time

	Estimate	95% CI	p-value
Enrollment Time (per 100 days)	0.16	(0.02, 0.29)	0.02
Discretized Enrollment Time^a^			0.04^b^
[0,33] days	Referent		
(33,119] days	−0.29	(−1.17, 0.60)	0.53
(119, 322] days	0.17	(−0.71, 1.04)	0.71
>322 days	0.97	(0.03, 1.91)	0.04
Sex			
Male	Referent		
Female	0.63	(0.06, 1.20)	0.03
Age			<0.001^b^
[50,60)	Referent		
[60,70)	−1.84	(−3.08, −0.60)	0.004
[70,80)	−2.63	(−3.81, −1.44)	<0.001
[80,90)	−4.09	(−5.31, −2.88)	<0.001
90+	−6.78	(−9.72, −3.83)	<0.001
Race & Ethnicity			0.06^b^
Non-Hispanic White	Referent		
Non-Hispanic Black	−0.62	(−1.95, 0.70)	0.36
Hispanic	−1.64	(−2.96, −0.32)	0.02
Asian/Pacific Islander	−1.80	(−4.16, 0.55)	0.13
Other Racio-Ethnic Group	1.04	(−1.77, 3.85)	0.47
Years of Education (per year)	0.15	(0.06, 0.25)	0.002
Baseline ADAS-Cog (per point)	0.05	(0.02, 0.09)	0.003

a. estimates obtained in an alternative model with enrollment time replaced by its discretization;

b. p-value obtained via a 2-sided multivariate Wald test of the hypothesis that coefficients corresponding to all factor levels are equal to zero.
