# Supplementary material for: Cohort Effects in Alzheimer’s Disease Trials: An Empirical Assessment Utilizing Data from the Alzheimer’s Disease Cooperative Study
Source: J Prev Alzheimers Dis. Author manuscript; Available in PMC 2026 Aug 19. (PMC13489100; doi:10.14283/jpad.2023.45)
Supplement: Supplementary file 1 [file NIHMS2130905-supplement-Supplementary_material.docx]

**Supplemental Material**

**Table S1:** Sample baseline demographics and 12-month change in ADAS-Cog by trial enrollment percentile.

|  | Enrollment Percentile | | | |
| --- | --- | --- | --- | --- |
|  | **[0,25]%** | **(25,50]%** | **(50-75]%** | **(75,100]%** |
| Sex, No. (%) |  |  |  |  |
| Female | 379 (55.6) | 360 (52.3) | 401 (57.5) | 394 (57.3) |
| Male | 303 (44.4) | 328 (47.7) | 296 (42.5) | 293 (42.7) |
| Age, No. (%) |  |  |  |  |
| [50,60) | 55 (8.1) | 50 (7.3) | 44 (6.3) | 55 (8.0) |
| [60,70) | 131 (19.2) | 134 (19.5) | 143 (20.5) | 137 (19.9) |
| [70,80) | 280 (41.1) | 288 (41.9) | 272 (39.0) | 298 (43.4) |
| [80,90) | 206 (30.2) | 206 (29.9) | 224 (32.1) | 189 (27.5) |
| 90+ | 10 (1.5) | 10 (1.5) | 14 (2.0) | 8 (1.2) |
| Race and Ethnicity, No. (%) |  |  |  |  |
| Non-Hispanic White | 617 (90.5) | 604 (87.8) | 608 (87.2) | 616 (89.7) |
| Non-Hispanic Black | 30 (4.4) | 36 (5.2) | 50 (7.2) | 31 (4.5) |
| Hispanic | 21 (3.1) | 33 (4.8) | 34 (4.9) | 28 (4.1) |
| Asian/Pacific Islander | 8 (1.2) | 8 (1.2) | 3 (0.4) | 7 (1.0) |
| Other | 6 (0.9) | 7 (1.0) | 2 (0.3) | 5 (0.7) |
| Study Partner Type^a^, No. (%) |  |  |  |  |
| Spouse | 430 (73.5) | 408 (69.2) | 389 (64.9) | 397 (67.2) |
| Adult Child | 113 (19.3) | 141 (23.9) | 153 (25.5) | 145 (24.5) |
| Other | 42 (7.2) | 41 (7.0) | 57 (9.5) | 49 (8.3) |
| Years of Education, mean (SD) | 14.5 (3.2) | 14.38 (3.0) | 14.33 (3.3) | 14.28 (3.2) |
| Baseline ADAS-Cog, mean (SD) | 24.6 (9.7) | 24.07 (9.6) | 24.65 (9.6) | 24.55 (10.3) |
| 12-Month ADAS-Cog Change, mean (SD) | 5.87 (7.8) | 6.08 (7.5) | 6.09 (7.3) | 5.53 (7.3) |
| % Completing Primary Endpoint | 74.8 | 75.4 | 73.9 | 78.8 |
| ^a^does not include participants from the IVIg trial | | | | |

**Table S2:** Summary of GEE regression of 12-month change in ADAS-Cog on trial enrollment percentile.

|  | Estimate | 95% CI | p-value |
| --- | --- | --- | --- |
| Enrollment Percentile (per 20%) | 0.12 | (-0.07, 0.32) | 0.22 |
| Discretized Enrollment Pctile.^a^ |  |  | 0.59^b^ |
| [0,25]% | Referent |  |  |
| (25,50]% | -0.21 | (-1.09, 0.66) | 0.63 |
| (50,75]% | 0.19 | (-0.67, 1.06) | 0.66 |
| (75,100]% | 0.33 | (-0.50, 1.16) | 0.44 |
| Sex |  |  |  |
| Male | Referent |  |  |
| Female | 0.62 | (0.04, 1.20) | 0.03 |
| Age |  |  | <0.001^b^ |
| [50,60) | Referent |  |  |
| [60,70) | -1.86 | (-3.09, -0.62) | 0.003 |
| [70,80) | -2.67 | (-3.85, -1.49) | <0.001 |
| [80,90) | -4.12 | (-5.33, -2.90) | <0.001 |
| 90+ | -6.85 | (-9.82, -3.88) | <0.001 |
| Race & Ethnicity |  |  | 0.06^b^ |
| Non-Hispanic White | Referent |  |  |
| Non-Hispanic Black | -0.65 | (-1.99, 0.70) | 0.34 |
| Hispanic | -1.59 | (-2.91, -0.28) | 0.02 |
| Asian/Pacific Islander | -1.83 | (-4.19, 0.52) | 0.13 |
| Other Racio-Ethnic Group | 1.02 | (-1.79, 3.82) | 0.48 |
| Years of Education (per year) | 0.15 | (0.06, 0.25) | 0.002 |
| Baseline ADAS-Cog (per point) | 0.06 | (0.02, 0.09) | 0.002 |
| ^a^estimates obtained in an alternative model with information proportion replaced by its discretization  ^a^p-value obtained via a 2-sided multivariate Wald test of the hypothesis that coefficients corresponding to all factor levels are equal to zero. | | | |

**Figure S1:** Summary of multinomial regression of study partner type on trial enrollment percentile. Left: summary of relative risk ratios of enrolling with an adult child study partner compared to a spousal one. Right: summary of relative risk ratios of enrolling with an other study partner compared to a spousal one. Within each panel the estimate corresponding to the model using continuous trial enrollment percentile is given on the left and those from the model using trial enrollment quartiles is given on the right.

**Figure S2:** Kaplan-Meier curves by trial enrollment quartiles. Legend: corresponding hazard ratio estimates and 95% CIs from a Cox proportional hazards model of time to study discontinuation on trial enrollment quartile; study completion or dying prior to primary endpoint completion were considered censoring. Bottom: Number of subjects at risk; cumulative number of dropout events given in paratheses.
